# Profiling the lncRNA-miRNA-mRNA ceRNA network to reveal potential crosstalk between inflammatory bowel disease and colorectal cancer

**DOI:** 10.7717/peerj.7451

**Published:** 2019-08-26

**Authors:** Fangfang Sun, Weiwei Liang, Kejun Tang, Mengying Hong, Jing Qian

**Affiliations:** 1 Cancer Institute (Key Laboratory of Cancer Prevention and Intervention, China National Ministry of Education, Key Laboratory of Molecular Biology in Medical Sciences, Zhejiang Province, China), The Second Affiliated Hospital, Zhejiang University School of Medicine, Hangzhou, Zhejiang, China; 2 Zhejiang University School of Medicine, Research Center of Infection and Immunity, ZJU-UCLA Joint Center for Medical Education and Research, Collaborative Innovation Center for Diagnosis and Treatment of Infectious Diseases, Hangzhou, Zhejiang, China; 3 Department of Endocrinology, The Second Affiliated Hospital, Zhejiang University School of Medicine, Hangzhou, China; 4 College of Pharmaceutical Sciences, Zhejiang University, Pharmaceutical Informatics Institute, Hangzhou, Zhejiang, China

**Keywords:** Inflammatory bowel disease, Colorectal cancer, Integrated bioinformatics, ceRNA network

## Abstract

**Background:**

Because of the increasing dysplasia rate in the lifelong course of inflammatory bowel disease (IBD) patients, it is imperative to characterize the crosstalk between IBD and colorectal cancer (CRC). However, there have been no reports revealing the occurrence of the ceRNA network in IBD-related CRC.

**Methods:**

In this study, we conducted gene expression profile studies of databases and performed an integrated analysis to detect the potential of lncRNA-miRNA-mRNA ceRNA in regulating disease transformation. R packages were used to screen differentially expressed mRNA, lncRNA and miRNA among CRC, IBD and normal tissue. The lncRNA-miRNA-mRNA network was constructed based on predicted miRNA-targeted lncRNAs and miRNA-targeted mRNAs. Functional analyses were then conducted to identify genes involved in the ceRNA network, and key lncRNAs were evaluated based on several clinical outcomes.

**Results:**

A total of three lncRNAs, 15 miRNAs, and 138 mRNAs were identified as potential mediators in the pathophysiological processes of IBD-related CRC. Gene Ontology annotation enrichment analysis confirmed that the dysplasia process was strongly associated with immune response, response to lipopolysaccharide, and inflammatory response. Survival analysis showed that LINC01106 (HR = 1.7; *p* < 0.05) were strongly associated with overall survival of colorectal cancer patients. The current study identified a series of IBD-related mRNAs, miRNA, and lncRNAs, and highlighted the important role of ceRNAs in the pathogenesis of IBD-related CRC. Among them, the LINC01106-miRNA-mRNA axis was identified as vital targets for further research.

## Introduction

Inflammatory bowel disease (IBD) includes ulcerative colitis (UC) and Crohn’s disease (CD), and is a chronic and incurable inflammatory disorder most frequently affecting the gastrointestinal tract. The main pathological changes of IBD involve intestinal fibrosis, abscesses, fistulas, and ulcers (Baumgart & Sandborn, 2012; Danese & Fiocchi, 2011). According to a report by Kappelman in 2007 in the United States, the prevalence of CD and UC in children <20 years of age was 43 and 28 per 100,000, respectively, while among adults the prevalence was 201 and 238 per 100,000, respectively (Kappelman et al., 2007). In Europe, the prevalence of IBD ranged from 3–7 cases for CD and 4–11 cases for UC per 100,000 (Vegh, Kurti & Lakatos, 2017). The prevalence has also been reported to vary widely in Asian countries (6.5–121 per 100,000) (Ng et al., 2013; Thia et al., 2008). The etiology and pathogenesis of IBD still remain unclear, and abnormal immune regulation, gut commensal microbes, gastrointestinal environmental factors, and genetic susceptibility are commonly cited as key factors (Khor, Gardet & Xavier, 2011). A recent study has also reported that IBD is still a global disease with an accelerating incidence in newly industrialized countries (Ng et al., 2018). Patients with IBD need a lifelong course of drugs, which results in immense financial and psychological burdens to patients and their families.

IBD is a significant risk factor for colorectal cancer (CRC). In 2001, by summarizing 116 studies of the relationships between IBD and CRC, Eaden, Abrams & Mayberry (2001) concluded that the overall prevalence of CRC was 3.7% in UC patients. Another meta-analysis reported the prevalence of UC-associated CRC as 0.85%, which was elevated to nearly 14% at 30 years in those with long-standing extensive colitis (Bopanna et al., 2017). Comparable findings have been demonstrated in CD and the reported incidence was 8% at 22 years. Similarly, the cumulative risk of CRC in CD was reported as 0.3%, 1.6%, and 2.4% at 5, 15, and 25 years after diagnosis (Shawki et al., 2018). Furthermore, IBD-associated CRC patients exhibit differences both in chemotherapeutic and clinical outcomes (Axelrad et al., 2017).

The competing endogenous RNA (ceRNA) hypothesis provides a better understanding of tumorigenesis (Salmena et al., 2011; Wee et al., 2012). LncRNAs contain miRNA-response elements, which function as ceRNAs, interacting with miRNAs to indirectly regulate mRNAs. The molecular pathogenesis of IBD-associated CRC has been widely reported in recent studies. Kameyama et al. (2018) reported similar genomic alterations between samples obtained from CRC and colitis-associated colorectal cancer, except for some distinct variations in the times and frequencies. Although an expanding number of miRNAs and lncRNAs have been shown to play a role in IBD-induced CRC, few of them reported comprehensive lncRNA-mRNA-miRNA ceRNA networks during disease development (Josse & Bours, 2016; Kanaan et al., 2012; Lewis et al., 2017; Svrcek et al., 2013; Zhu et al., 2016).

In the present study, we identified a potential ceRNA network and characterized the potential mechanism during IBD-related CRC using bioinformatic analysis. We examined differentially expressed genes (DEGs) among CRC (or IBD related neoplasia), IBD and normal tissue based on three previously published gene datasets, GSE10714, GSE4183 and GSE68306, available in the Gene Expression Omnibus (GEO) database. By taking full advantage of The Cancer Genome Atlas (TCGA, https://portal.gdc.cancer.gov), an authoritative dataset COAD was used to identify abnormally expressed mRNAs, lncRNAs, and miRNAs in CRC compared to normal tissue. Together with four datasets, the crosstalk network among aberrant expressions of lncRNA-miRNA-mRNA was constructed to predict candidate diagnostic biomarkers and therapeutic targets for IBD-induced CRC. Further functional analyses, including Gene Ontology (GO) and the Kyoto Encyclopedia of Genes and Genomes (KEGG) pathway enrichment, protein-protein interaction (PPI) network, transcription factor (TF) enrichment, and survival analysis were performed to explore potentially important genes involved in the transformation from IBD to CRC.

## Materials & Methods

### Expression profile dataset

**Figure 1:**
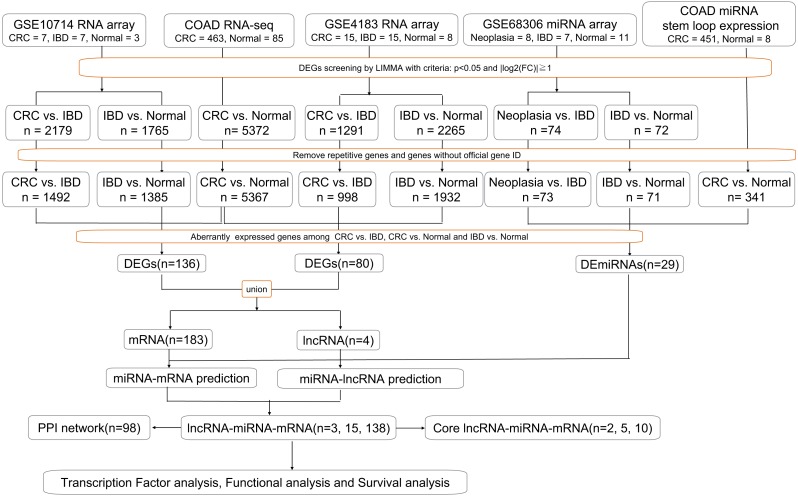
Workflow of our bioinformatics analyses. In the figure, “n” means number of genes.

Gene expression profiles for IBD-related CRC (GSE10714, GSE4183 and GSE68306) were obtained from the GEO database. GSE10714 and GSE4183 were normalized genome-wide gene expression profiles, evaluated by HGU133 Plus 2.0 microarrays. In GSE4183, the RNA expression profiles of 15 patients with CRC, 15 with IBD, and eight healthy normal controls were included. In GSE10714, seven patients with CRC, seven with IBD (four with CD and three with UC), and three healthy normal controls were chosen. Meanwhile, GSE68306 is a miRNA array, in which samples were grouped into normal tissue (*n* = 16), UC without neoplasia (UC, *n* = 9), and UC-associated neoplastic tissue (*n* = 11). The quality of normalized gene expression data was analyzed and visualized using the ggplot2 package of R software for each group and sample. Moreover, COAD RNA-seq dataset was analyzed online by GEPIA based on TCGA database which contained 463 tumor tissue and 85 normal tissue (two recurrent and one metastasis tissue have been excluded). COAD miRNA expression profile containing 451 tumor sample and eight normal samples (one recurrent and one metastasis samples have been excluded) was obtained from XENA (a portal website for TCGA) and analyzed in R. All of the following processes have been shown in flow chart (Fig. 1).

### Differential gene expression analyses

We analyzed the CRC/Neoplasia vs IBD groups and IBD vs Normal groups to identify the DEGs in GSE10714 and GSE4183, and differential expressed miRNAs (DE-miRNA) in GSE68306 by utilizing The Linear Models for Microarray data (LIMMA) package (Ritchie et al., 2015), which included lmFit, eBayes, and topTable functions. *P* < 0.05 and — log2-fold change —>1 were used as the cut-off criteria. The DEG screening between tumor tissue and normal tissue in COAD dataset was performed using the GEPIA (Tang et al., 2017) website with the same criteria by using LIMMA method, and their transcript biotypes were annotated using the bioMart package in R. DE-miRNAs in CRC vs Normal were screened by LIMMA in R with the same criteria as before based on COAD miRNA stem loop expression profile.

### Constructing the ceRNA network

Along with identified differential expressed mRNAs, lncRNAs and miRNA, we constructed the ceRNA network as follows. 1. To predict the miRNA-targeted lncRNAs, we used DIANA-LncBase version 2 (http://carolina.imis.athena-innovation.gr/), starBase (http://starbase.sysu.edu.cn/) (Li et al., 2014; Yang et al., 2011), and RAID version 2.0 (http://www.rna-society.org/raid/) (Yi et al., 2016) to analyze interactions between lncRNAs and miRNAs. 2. To identify the miRNA-targeted mRNAs, we used DIANA-TarBase version 8 (http://carolina.imis.athena-innovation.gr/) and TargetScan (http://www.targetscan.org/). 3. To construct and visualize the LncRNA-miRNA-mRNA network, Cytoscape 3.7.0 software was used to construct and visualize the ceRNA network as well as its core network according to synergic expression of mRNA and lncRNA.

### Functional analyses

GO annotation, including molecular function, cell component and biological process, and KEGG, summarizing genomes, biological pathways and health information, were used to clarify the potential role of the DEGs in the ceRNA network. The functional analysis was conducted using DAVID (Database for Annotation, Visualization and Integrated Discovery, version 6.8) (Huang, Sherman & Lempicki, 2008a; Huang, Sherman & Lempicki, 2008b), with *P* < 0.05 used as a cut off. The upstream transcription factors enriched for these DEGs were calculated using the UCSC Genome Browser (http://genome.ucsc.edu/). We constructed a PPI network for the DEGs using STRING (Szklarczyk et al., 2018), an online functional protein association network tool, to detect potential relationships among the DEGs with confidence scores ≥ 0.4 and a maximum number of interactors of one. The CentiScaPe plugin (Scardoni et al.) was used to determine the characteristics of each node in the PPI network, which assigned each gene a degree score, the simplest topological index, allowing for immediate evaluation of the average number of edges (interactions) incident to the node.

### Survival analysis

Because lncRNAs listed in the ceRNA network can potentially regulate mRNA expression by sponging related miRNA, they are identified as pivotal genes in the network. LncRNAs correlations with stage expressions and patient survival were featured in GEPIA based on available TCGA patient survival data incuding COAD and READ datasets and Kaplan–Meier survival analysis is applied to generate overall and disease-free survival plots online.

### Statistical analysis

Statistical analyses were performed using R studio. Expression Data were shown as mean ± SD. Paired sample t test, one-way ANOVA test, and the rank-sum test were flexibly used in different experiments. A 2-tailed *P* value less than 0.05 was considered to be statistical significant.

## Results

### Data distribution analyses and DEG screening

**Figure 2:**
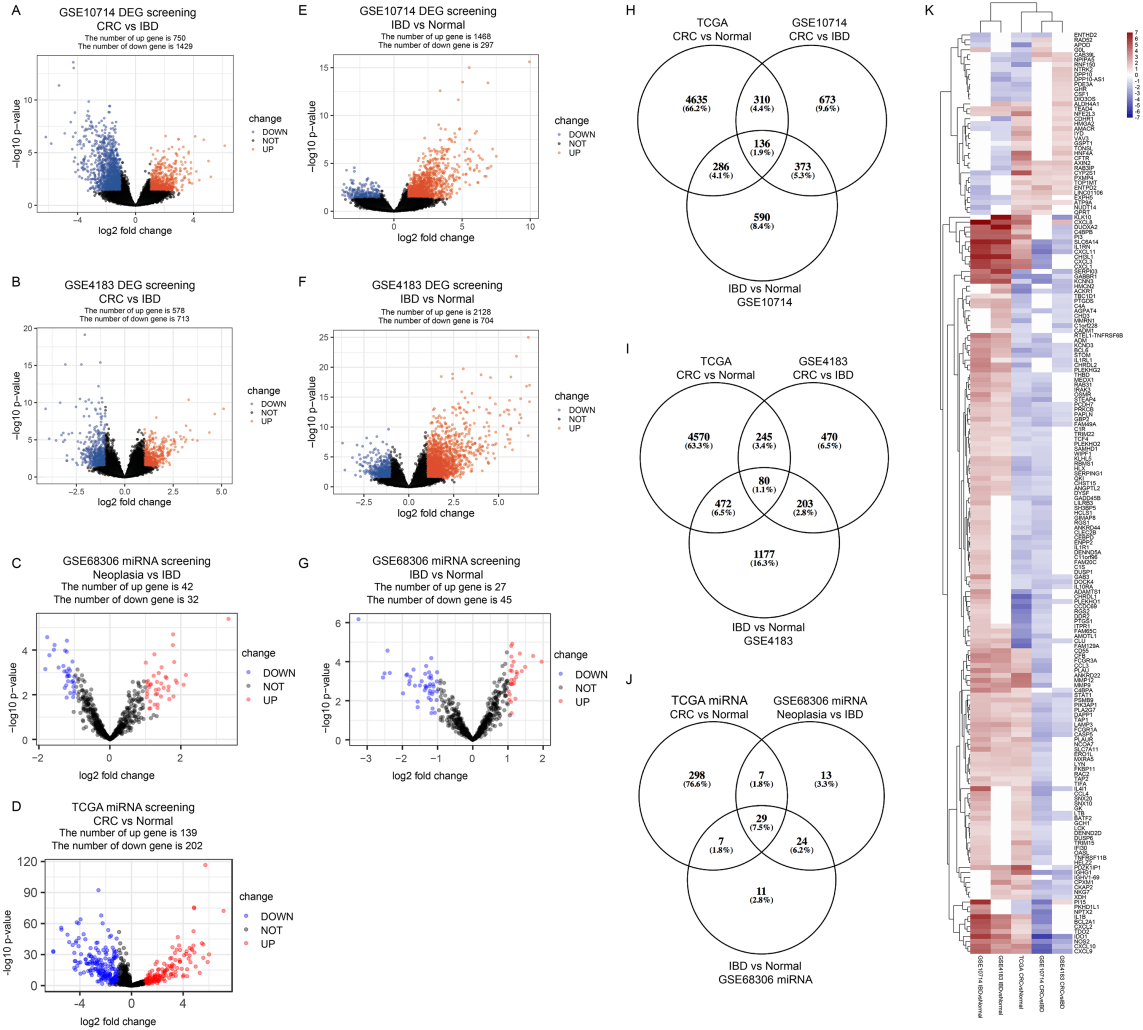
Identify differential expressed genes in the disease progression from IBD to CRC. (A–G) Volcano graph displaying pairs of expressed genes. (H) Venn analysis among DEGs of CRC vs. IBD in GSE10714, IBD vs. Normal in GSE10714, GSE4183, and CRC vs Normal in TCGA. (I) Venn analysis among DEGs of CRC vs. IBD in GSE4183, IBD vs. Normal in GSE4183, and CRC vs. Normal in TCGA. (J) Venn analysis among DE-miRNAs of CRC vs. IBD in GSE68306, IBD vs. Normal in GSE68306, and CRC vs. Normal in TCGA miRNA. (K) Heatmap and hierarchical clustering of identified 187 DEGs. Up or down regulated genes are colored in red or blue, respectively.

In GSE10714 and GSE4183, a total of 54,675 genes were detected in 17 samples. The expression values and the distributions were similar between the three groups in each dataset (Figs. S1A, and S1D for GSE10714; Figs. S1B, and S1E for GSE4183). These data distribution analyses showed that the two GEO datasets were qualified for further bioinformatics analyses. In GSE68306 miRNA array dataset, a total of 654 miRNAs were detected in 36 samples. Among them, samples Normal 12-16, IBD 8-9, and Neoplasia 9-11 were ruled out because data distribution of these samples were quite different from others in the same group (Fig. S1F). The expression density of three groups in GSE68306 is similar after filtration (Fig. S1C). Following data processing using LIMMA, we identified 2,179 DEGs, 1,291 DEGs, and 74 DE-miRNAs in the CRC/Neoplasia group vs. the IBD group for GSE10714 (Fig. 2A), GSE4183 (Fig. 2B) and GSE68306 (Fig. 2C), respectively; meanwhile, 1765 DEGs, 2265 DEGs and 72 DE-miRNAs were identified in the IBD group vs. the Normal group for GSE10714 (Fig. 2E), GSE4183 (Fig. 2F) and GSE68306 (Fig. 2G), respectively. According to GEPIA, 5,372 DEGs in the CRC group vs. the Normal group were obtained with the same cut-off criteria. Also, 341 DE-miRNAs in the CRC group vs. the Normal group were identified by R based on COAD miRNA expression profile (Fig. 2D). Repetitive genes with different probes and these predicted genes without official gene symbol or transcript annotation in NCBI or Ensembl were removed in the following analysis.

### Disease progression associated DEGs

In our study, we meant to compare the differences between IBD and CRC to figure out potential genomic change that contribute to the disease progression from IBD to CRC. Also, we need to make sure these DEGs are both significantly differential expressed in CRC vs. Normal comparison and IBD vs. Normal comparison which make sense that they are involved in the development of tumorigenesis. By intersection using three comparisons (CRC/Neoplasia vs. IBD, IBD vs. Normal and CRC vs Normal) and combining data in two intersections(Figs. 2H and 2I), we identified a total of 187 DEGs as the IBD-related DEGs, including four lncRNAs and 183 mRNAs. Hierarchical clustering of the identified DEGs is displayed as a heat map to show the gene clusters sharing similar expression change among CRC, IBD and normal tissue (Fig. 2K) which indicated that most of DEGs are higher expressed in IBD than CRC. By intersection using three comparisons of miRNA data, 29 miRNAs are recognized (Fig. 2J). The information for these DEGs, DE-miRNAs and heatmap expression data was shown in Tables S1–S9.

### Construction of the ceRNA network

**Figure 3:**
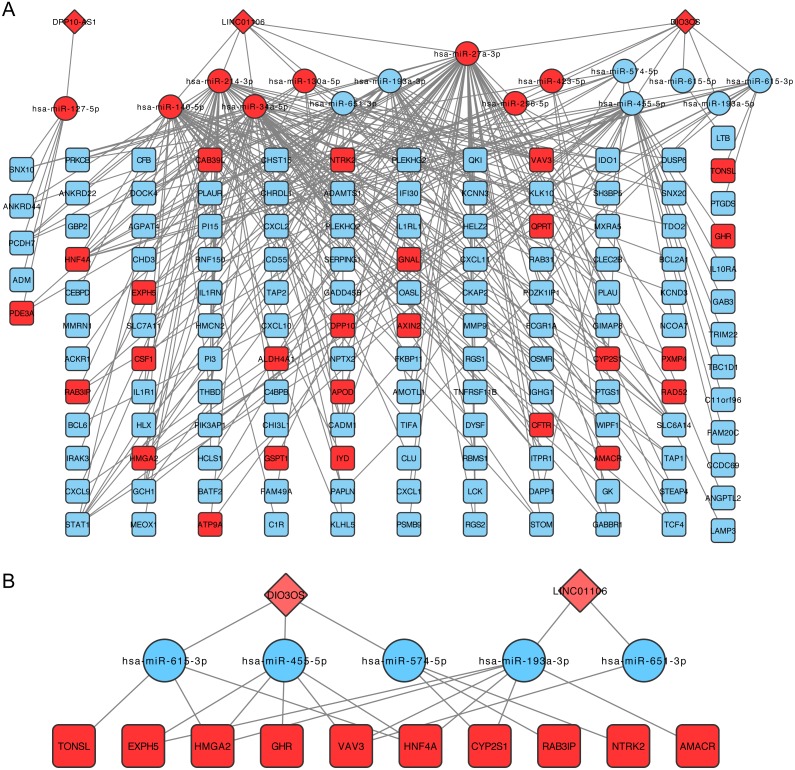
lncRNA-miRNA-mRNA ceRNA network. (A) is the total ceRNA network and (B) is the core ceRNA network containing synergic expressed mRNA and lncRNA but divergent expressed miRNAs. Diamonds stand for lncRNAs, rectangles stand for mRNAs, and ellipses stand for mRNA. Up or down regulated genes are colored in red or blue, respectively.

The lncRNA-miRNA-mRNA network was then constructed following the steps listed in the Materials and Methods, which contained three lncRNA, 15 miRNAs, and 138 mRNA (Fig. 3A). CeRNA is said to act as a sponge for miRNAs and free mRNA from miRNA binding. Therefore, a core ceRNA network was extracted including synergic expressed mRNA and lncRNA but divergent expressed miRNAs, and shown in Fig. 3B. These three lncRNAs in Fig. 3A were termed key ceRNAs. The interactions and nodes information in ceRNA network were shown in Tables S10 and S11, respectively.

### Functional analyses

**Figure 4:**
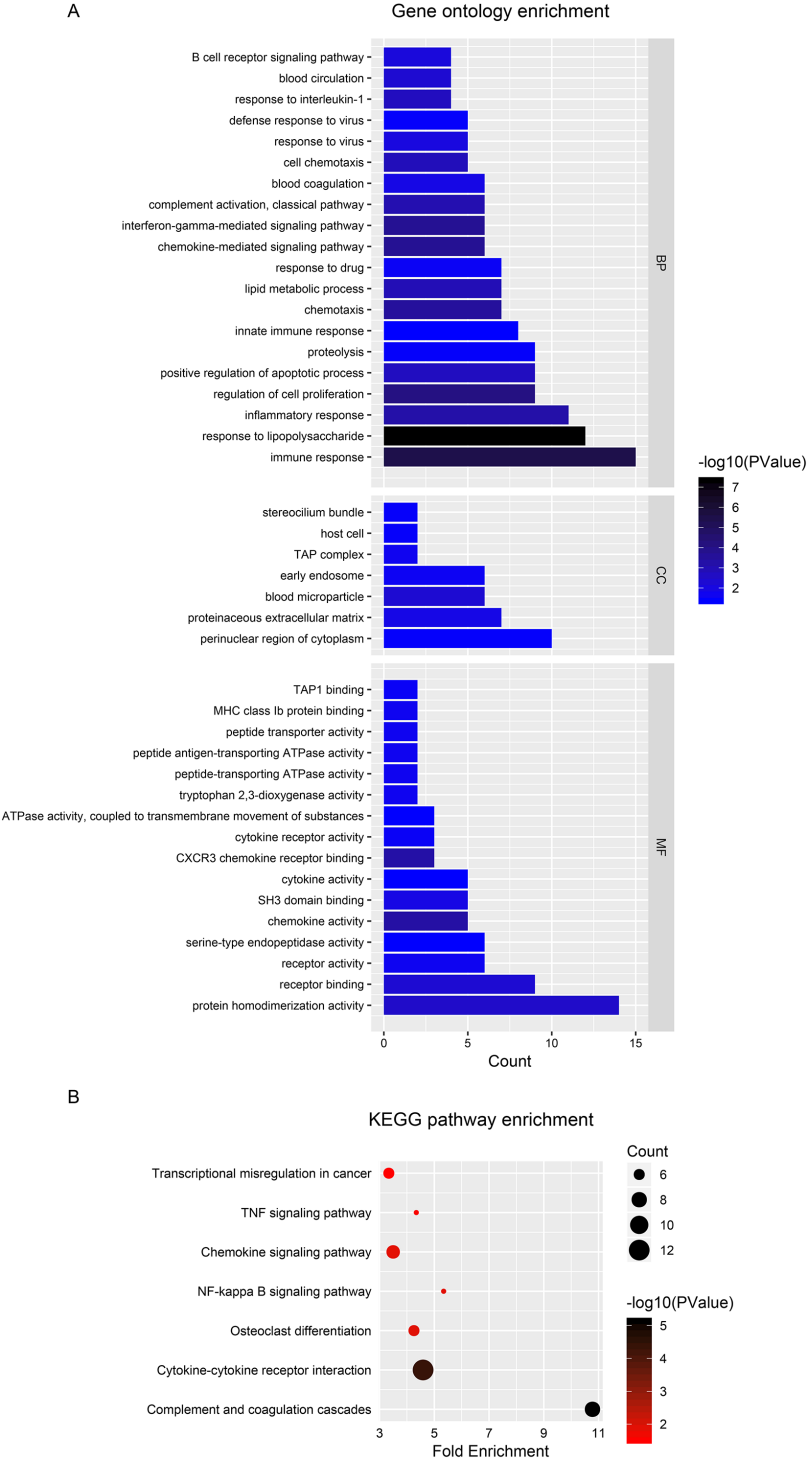
Functional analysis of DEGs in ceRNA network. (A) Histogram of Gene Ontology (GO) functional classification of DEGs. The *x*-axis represents the number of DEGs, with individual GO terms plotted on the *y*-axis. All GO terms were grouped into three categories: biological processes, cellular components, and molecular functions. The graph displays only significantly enriched GO terms (*P* < 0.05), with darker blue indicating greater significance. (B) Histogram of Kyoto Encyclopedia of Genes and Genomes (KEGG) pathways enrichment in DEGs. The *x*-axis represents the number of DEGs annotated in a pathway, with individual KEGG terms shown on the *y*-axis. The graph displays only significantly enriched KEGG terms (*P* < 0.05), with darker red indicating greater significance.

To explore the functional significance and potential regulators of the mRNAs involved in the ceRNA network, GO annotation and KEGG pathway enrichment analyses were conducted. Figure 4A shows that ceRNAs may affect the transformation from IBD to CRC by regulating the immune response, response to lipopolysaccharide and inflammatory response in biological processes. Significant cellular component terms revealed that proteins coded by these mRNAs were mainly located in the perinuclear region of cytoplasm. The top three molecular functions identified were protein homodimerization, receptor binding, and receptor activity. As for KEGG pathway enrichment, the ceRNA relevant mRNAs were mostly enriched in complement and coagulation cascades, cytokine-cytokine receptor interaction, and osteoclast differentiation (Fig. 4B). The detailed genes involved in each term are listed in the Tables 1 and 2.

**Figure 5:**
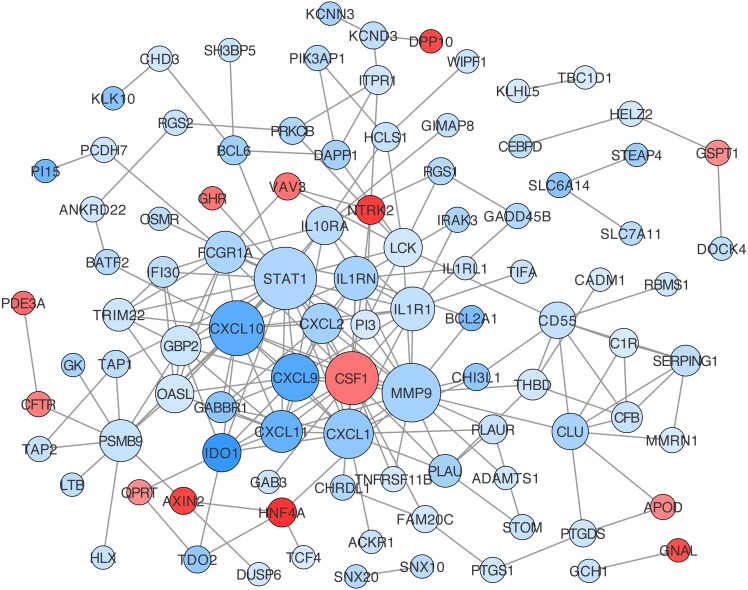
Protein-protein interaction (PPI) network construction for DEGs in ceRNA network. The value of centrality degree is marked by different node size. Up or down regulation of genes according to CRC vs. IBD comparison are filled with red or blue color, respectively.

Construction of protein-protein interaction networks among these genes was conducted based on String in Cytoscape. The centrality degree of each node was evaluated by the CentiScape plugin. Twenty-six genes were defined as hub genes with the criterion, degree >5, including *STAT1, MMP9, CXCL10, CSF1, CXCL1, CXCL9, IL1R1, IL1RN, CXCL11, PSMB9* and so on (Table S12). Among them, *STAT1, MMP9*, and *CXCL10* were the top three nodes centralized in net with the largest size (Fig. 5).

**Figure 6:**
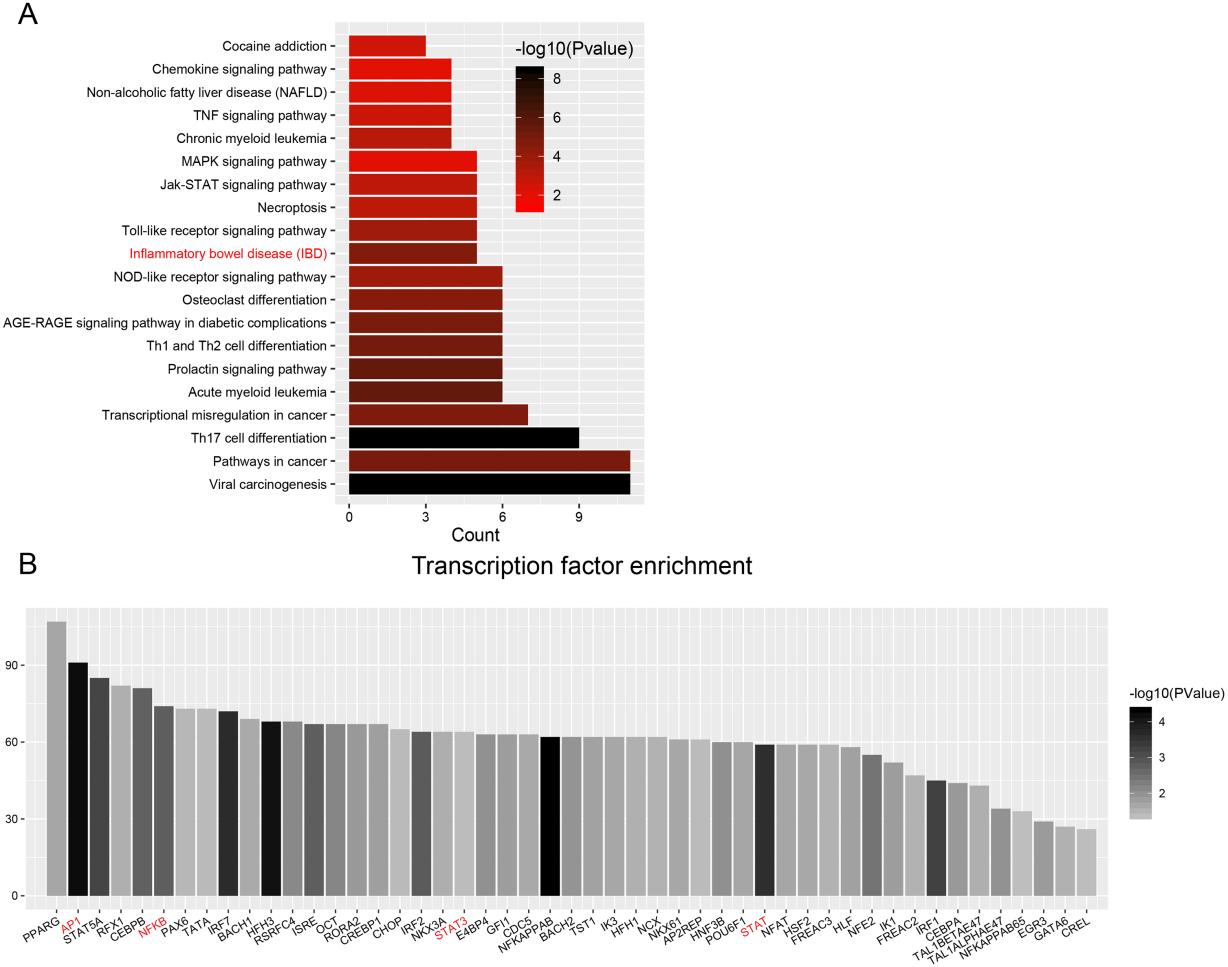
Transcription factor enrichment. (A) KEGG pathway enrichment for enriched TFs. The graph displays only significantly enriched KEGG terms (*P* < 0.05), with darker red indicating greater significance. (B) Histogram of TFs shows the count of genes downstream each TF.

Enrichment analyses of transcription factors (TFs) of upstream DEGs in the ceRNA network indicated that inflammatory bowel disease (JUN, NFKB1, STAT1, STAT3 and GATA6) and pathways in cancer (CCDC6, CEBPA, JUN, NFKB1, NKX31, PPARG, RUNX1, STAT1, STAT3, STAT5A, and STAT5B) were included in the KEGG pathway list (Fig. 6A and Table 3). The number of DEGs in each TF is shown as a histogram in Fig. 6B. JUN (also known as AP1) and NFKB1 regulated more than 50% of the genes in the list. The DEGs targeted by TFs were listed in Table S13.

### Survival analysis for ceRNA

**Figure 7:**
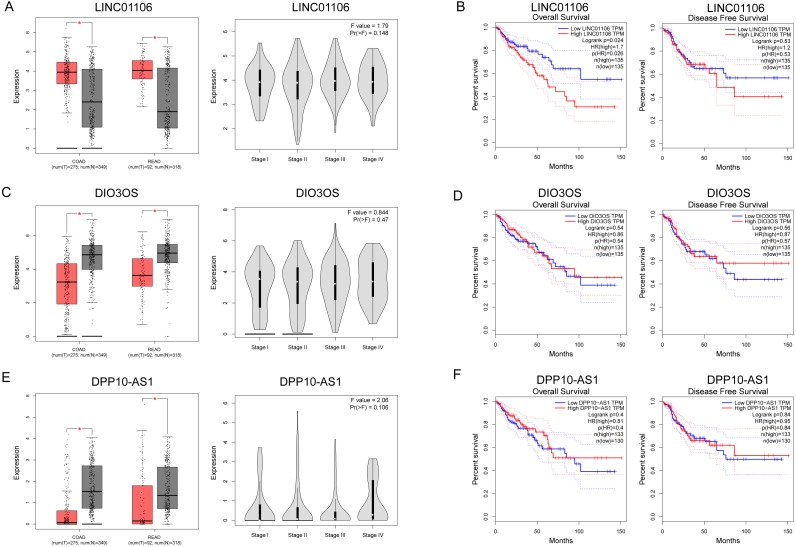
Clinical significance of three key lncRNAs in ceRNA network. (A, C & E) Analyses of key lncRNAs expression in tumor vs. normal tissues and in different tumor stages. Red box stands for tumor tissue; grey box is for normal tissue; and dots presents each sample value in left panel. (B, D & F) Overall and disease-free survival analyses of key lncRNAs. *P < 0.05; other *P*-values are shown on the diagrams. READ, rectum adenocarcinoma; COAD, colon adenocarcinoma.

The expressions of three ceRNA from the TCGA dataset were plotted to reveal differences among Stages I–IV (Figs. 7A, 7C and 7E). None of them was significantly differently expressed in the higher stage (*p* > 0.05). Survival analysis showed that LINC01106 (Fig. 7B; HR = 1.7; *p* < 0.05) was strongly associated with overall survival of CRC patients.

## Discussion

In the current study, we used bioinformatic analysis to identify IBD-related CRC mRNAs and lncRNAs based on the published database obtained from the GEO and TCGA. By constructing a lncRNA-miRNA-mRNA network, a total of three lncRNAs, 15 miRNAs, and 138 mRNAs were identified as potential mediators in the pathophysiological processes of IBD-related CRC. Moreover, we also performed functional analyses to investigate the potential biological functions and pathways involved in the disease progression from IBD to CRC.

**Table 1:** Information of GO enrichment for DEGs.

Category	Term	Count	*P* value	Genes
BP	Immune response	15	3.68E-06	CXCL1, IL1R1, IL1RL1, IL1RN, CXCL2, CXCL9, C1R, CXCL11, TRIM22, CXCL10, TNFRSF11B, RGS1, FCGR1A, LTB, GBP2
BP	Response to lipopolysaccharide	12	4.62E-08	CXCL1, IRAK3, TNFRSF11B, THBD, ADM, IL10RA, CXCL2, CXCL9, IDO1, CXCL11, CXCL10, GCH1
BP	Inflammatory response	11	6.13E-04	CXCL1, TNFRSF11B, CSF1, CXCL2, PTGS1, ACKR1, CHI3L1, CXCL9, BCL6, CXCL11, CXCL10
BP	Regulation of cell proliferation	9	8.19E-05	TNFRSF11B, CXCL2, LCK, PTGS1, CXCL9, BCL6, CXCL11, PLAU, CXCL10
BP	Positive regulation of apoptotic process	9	2.02E-03	PLEKHG2, VAV3, ADM, BCL2A1, CLU, BCL6, GADD45B, HMGA2, DUSP6
BP	Proteolysis	9	3.62E-02	IGHG1, CFB, MMP9, DPP10, ADAMTS1, KLK10, PAPLN, C1R, PLAU
BP	Innate immune response	8	4.50E-02	IGHG1, CD55, CSF1, LCK, CLU, SERPING1, C4BPB, C1R
BP	Chemotaxis	7	3.28E-04	CXCL1, CXCL2, CXCL9, CXCL11, PLAU, CXCL10, PLAUR
BP	Lipid metabolic process	7	1.24E-03	APOD, HNF4A, PTGDS, PTGS1, CLU, IL1RN, PDE3A
BP	Response to drug	7	2.81E-02	TNFRSF11B, VAV3, APOD, LCK, PDE3A, STAT1, DUSP6
BP	Chemokine-mediated signaling pathway	6	2.00E-04	CXCL1, CXCL2, ACKR1, CXCL9, CXCL11, CXCL10
BP	Interferon-gamma-mediated signaling pathway	6	2.00E-04	OASL, FCGR1A, IFI30, STAT1, TRIM22, GBP2
BP	Complement activation, classical pathway	6	9.27E-04	IGHG1, CD55, CLU, SERPING1, C4BPB, C1R
BP	Blood coagulation	6	1.30E-02	THBD, HNF4A, C4BPB, MMRN1, PLAU, PLAUR
BP	Cell chemotaxis	5	1.48E-03	CXCL1, CXCL2, CXCL9, DOCK4, CXCL10
BP	Response to virus	5	9.73E-03	IRAK3, OASL, CLU, HMGA2, TRIM22
BP	Defense response to virus	5	3.67E-02	OASL, CXCL9, STAT1, TRIM22, CXCL10
BP	Response to interleukin-1	4	1.95E-03	IRAK3, IL1R1, CHI3L1, GHR
BP	Blood circulation	4	4.76E-03	ADM, SERPING1, STAT1, CXCL10
BP	B cell receptor signaling pathway	4	7.91E-03	IGHG1, VAV3, LCK, PRKCB
CC	Perinuclear region of cytoplasm	10	3.68E-02	STOM, LAMP3, APOD, PTGDS, CSF1, ATP9A, CLU, CHI3L1, STA1, GBP2
CC	Proteinaceous extracellular matrix	7	1.34E-02	TNFRSF11B, IL1RL1, MMP9, PI3, CHI3L1, ADAMTS1, PAPLN
CC	Blood microparticle	6	5.01E-03	IGHG1, STOM, CFB, CLU, SERPING1, C1R
CC	Early endosome	6	2.57E-02	RAB31, LAMP3, DYSF, ATP9A, ACKR1, CFTR
CC	TAP complex	2	2.16E-02	TAP2, TAP1
CC	Host cell	2	3.57E-02	TAP2, TAP1
CC	Stereocilium bundle	2	3.57E-02	IDO1, DOCK4
MF	Protein homodimerization activity	14	3.17E-03	CADM1, CEBPD, CSF1, BCL2A1, STAT1, GCH1, STOM, IRAK3, HNF4A, TAP1, NTRK2, QPRT, TCF4, GHR
MF	Receptor binding	9	5.01E-03	CXCL1, ADM, CADM1, HNF4A, AMACR, ANGPTL2, LTB, CXCL10, PLAUR
MF	Receptor activity	6	2.34E-02	TNFRSF11B, THBD, CADM1, IL10RA, ACKR1, PLAUR
MF	Serine-type endopeptidase activity	6	4.24E-02	IGHG1, CFB, MMP9, KLK10, C1R, PLAU
MF	Chemokine activity	5	4.84E-04	CXCL1, CXCL2, CXCL9, CXCL11, CXCL10
MF	SH3 domain binding	5	1.22E-02	SH3BP5, HCLS1, QKI, WIPF1, DOCK4
MF	Cytokine activity	5	4.29E-02	TNFRSF11B, CSF1, IL1RN, CXCL9, LTB
MF	CXCR3 chemokine receptor binding	3	5.45E-04	CXCL9, CXCL11, CXCL10
MF	Cytokine receptor activity	3	2.95E-02	IL1RL1, OSMR, GHR
MF	ATPase activity, coupled to transmembrane movement of substances	3	4.26E-02	TAP2, TAP1, CFTR
MF	Tryptophan 2,3-dioxygenase activity	2	2.22E-02	TDO2, IDO1
MF	Peptide-transporting ATPase activity	2	2.22E-02	TAP2, TAP1
MF	Peptide antigen-transporting ATPase activity	2	2.22E-02	TAP2, TAP1
MF	Peptide transporter activity	2	2.22E-02	TAP2, TAP1
MF	MHC class Ib protein binding	2	2.22E-02	TAP2, TAP1
MF	TAP1 binding	2	2.95E-02	TAP2, TAP1

**Table 2:** Information of KEGG pathway enrichment for DEGs.

Number	Term	Count	Fold enrichment	*P* value	Genes
hsa04610	Complement and coagulation cascades	8	10.78	7.23E-06	CD55, THBD, CFB, SERPING1, C4BPB, C1R, PLAU, PLAUR
hsa04060	Cytokine-cytokine receptor interaction	12	4.59	4.36E-05	CXCL1, IL1R1, TNFRSF11B, OSMR, IL10RA, CSF1, CXCL2, CXCL9, CXCL11, LTB, GHR, CXCL10
hsa04380	Osteoclast differentiation	6	4.26	1.24E-02	IL1R1, TNFRSF11B, FCGR1A, CSF1, LCK, STAT1
hsa04064	NF-kappa B signaling pathway	5	5.34	1.34E-02	IL1R1, LCK, BCL2A1, LTB, PLAU
hsa04062	Chemokine signaling pathway	7	3.50	1.37E-02	CXCL1, VAV3, CXCL2, CXCL9, CXCL11, STAT1, CXCL10
hsa04668	TNF signaling pathway	5	4.34	2.65E-02	CXCL1, CSF1, MMP9, CXCL2, CXCL10
hsa05202	Transcriptional misregulation in cancer	6	3.34	3.17E-02	FCGR1A, MMP9, BCL2A1, BCL6, HMGA2, PLAU

**Table 3:** KEGG pathway enrichment for transcription factors.

KEGG Term	Count	*P* value	TFs
Viral carcinogenesis	11	3.6E-09	ATF2,EGR3,IRF3,IRF7,IRF9,JUN,NFKB1,STAT3,STAT5A,STAT5B,TBP
Pathways in cancer	11	1.2E-05	CCDC6,CEBPA,JUN,NFKB1,NKX3-1, PPARG, RUNX1, STAT1, STAT3, STAT5A, STAT5B
Th17 cell differentiation	9	3.6E-09	GATA3,IRF4,JUN,NFKB1,RUNX1,STAT1,STAT3,STAT5A,STAT5B
Transcriptional misregulation in cancer	7	1.7E-05	CEBPA,CEBPB,DDIT3,NFKB1,PPARG,RUNX1,ZEB1
Acute myeloid leukemia	6	2.9E-06	CEBPA,NFKB1,RUNX1,STAT3,STAT5A,STAT5B
Prolactin signaling pathway	6	3.1E-06	IRF1,NFKB1,STAT1,STAT3,STAT5A,STAT5B
Th1 and Th2 cell differentiation	6	8.8E-06	GATA3,JUN,NFKB1,STAT1,STAT5A,STAT5B
AGE-RAGE signaling pathway in diabetic complications	6	1.3E-05	JUN,NFKB1,STAT1,STAT3,STAT5A,STAT5B
Osteoclast differentiation	6	2.9E-05	BLNK,IRF9,JUN,NFKB1,PPARG,STAT1
NOD-like receptor signaling pathway	6	1.3E-04	IRF3,IRF7,IRF9,JUN,NFKB1,STAT1
Inflammatory bowel disease (IBD)	5	2.3E-05	GATA3,JUN,NFKB1,STAT1,STAT3
Toll-like receptor signaling pathway	5	1.6E-04	IRF3,IRF7,JUN,NFKB1,STAT1
Necroptosis	5	9.1E-04	IRF9,STAT1,STAT3,STAT5A,STAT5B
Jak-STAT signaling pathway	5	9.7E-04	IRF9,STAT1,STAT3,STAT5A,STAT5B
MAPK signaling pathway	5	9.2E-03	ATF2,DDIT3,JUN,NF1,NFKB1
Chronic myeloid leukemia	4	7.4E-04	NFKB1,RUNX1,STAT5A,STAT5B
TNF signaling pathway	4	2.3E-03	ATF2,CEBPB,JUN,NFKB1
Non-alcoholic fatty liver disease (NAFLD)	4	6.0E-03	CEBPA,DDIT3,JUN,NFKB1
Chemokine signaling pathway	4	9.7E-03	NFKB1,STAT1,STAT3,STAT5B
Cocaine addiction	3	2.8E-03	ATF2,JUN,NFKB1
Cytosolic DNA-sensing pathway	3	5.1E-03	IRF3,IRF7,NFKB1
Non-small cell lung cancer	3	5.9E-03	STAT3,STAT5A,STAT5B
RIG-I-like receptor signaling pathway	3	6.5E-03	IRF3,IRF7,NFKB1
B cell receptor signaling pathway	3	6.5E-03	BLNK,JUN,NFKB1
Pancreatic cancer	3	6.9E-03	NFKB1,STAT1,STAT3
ErbB signaling pathway	3	9.2E-03	JUN,STAT5A,STAT5B
Longevity regulating pathway	3	9.9E-03	ATF2,NFKB1,PPARG
IL-17 signaling pathway	3	1.1E-02	CEBPB,JUN,NFKB1
Prostate cancer	3	1.2E-02	NFKB1,NKX3-1,ZEB1
Relaxin signaling pathway	3	2.5E-02	ATF2,JUN,NFKB1
Fluid shear stress and atherosclerosis	3	2.6E-02	JUN,MEF2A,NFKB1
Apoptosis	3	2.6E-02	DDIT3,JUN,NFKB1
Maturity onset diabetes of the young	2	1.1E-02	FOXA2,PAX6
Thyroid cancer	2	1.9E-02	CCDC6,PPARG
Amphetamine addiction	2	4.6E-02	ATF2,JUN
Adipocytokine signaling pathway	2	5.0E-02	NFKB1,STAT3

According to our GO annotation results, DEGs included in ceRNA network, could regulate the IBD-related CRC through involvement in the regulation of the immune response, response to lipopolysaccharide (LPS), and inflammation. The immune response was shown to play an important role in the pathogenesis of IBD. LPS was well known as a TLR4 activator (Chowdhury, Sacks & Sheerin, 2006). As an important mediator involved in the host immune response, TLR4 is overexpressed in inflammation-associated colorectal neoplasia tissues, and contributes to the production of inflammatory factors in chronic colitis. Moreover, TLR4-deficient mice exhibit lower rates of colonic polyps (Fukata et al., 2007). Together, these results showed a critical role for the TLR4-mediated immune response in the tumorigenesis of colitis-associated CRC. Conversely, Lowe et al. reported that mice with colitis-associated colorectal cancer deficient in TLR2 easily developed significantly more and larger colorectal tumors compared with wild-type mice, together with increased expressions of IL-6, IL-17A, and phospho-STAT3 (Lowe et al., 2010). Inflammation was also widely reported in IBD-related CRC. Necrosis and regeneration of intestinal mucosa were the main pathological changes of IBD. Longer-duration chronic inflammation in IBD could lead to the abnormal regulation of the immune system (Kuraishy, Karin & Grivennikov, 2011). A number of proinflammatory pathways have been recently identified in inflammation-associated tumor development (Long, Lundsmith & Hamilton, 2017). Specific molecular signaling pathways such as NF-κB, has been implicated in the carcinogenesis of IBD-related CRC. NF-κB has been identified as a key upstream TF in regulating DEGs from ceRNA and NF-κB related pathway, a downstream part of LPS/TLR4, is highly enriched and popped up in KEGG pathway enrichment analysis. Deletion of IKKβ in intestinal epithelial cells decreased the incidence of colitis-associated cancer in the colitis-associated mouse cancer model (Greten et al., 2004). IL-6/STAT3 was also demonstrated to be involved in metabolic reprogramming, to promote the progression from chronic colitis to CRC (Qu et al., 2017), and the COX-2/PGE2 pathway was also shown to contribute to inflammation and carcinogenesis in IBD. Overexpression of COX-2 was observed in UC-associated neoplasia at both the gene and protein levels (Agoff et al., 2000). Peroxisome proliferator-activated receptor δ (PPARδ) was shown to exert an important role in chronic inflammation and pathophysiological processes of CRC via the COX-2/PGE2 pathway (Wang & DuBois, 2010; Wang et al., 2014). *In vivo* studies showed that PPAR *δ* was necessary for the production of proinflammatory mediators, such as chemokines, cytokines, COX-2, and PGE2, which led to colitis-associated tumor growth (Wang et al., 2014). In general, these immune response and inflammatory response-related hub genes identified in our study, including *CXCL10, CSF1, CXCL1, CXCL9, IL1R1, IL1RN, CXCL11, FCGR1A, CXCL2* and *GBP2* (centrality degree >10), deserve further research and discussion. Interestingly, all these genes are downregulated in CRC vs. IBD group, while they are all higher expressed in CRC and IBD compared to normal tissue. It seems that they are dynamically regulated according to the necessity of tumorigenesis process. Further research need be done to indicate the potential function of these genes in IBD related CRC.

We also identified three key ceRNAs, DPP10-AS1, DIO3OS and LINC01106, which were not ascertained in previous studies. Among them, LINC01106 is the only ceRNA related to overall survival of CRC patients. LIN01106 contained a transcript of 2204 bp with two exons located on chromosome 2q13. However, few studies have been conducted on the function of LINC01106. Our results showed intimate interactions between LINC01106 and miRNAs, such as MIR651, MIR193A, MIR140, MIR214, MIR34A, and MIR130A. Among them, MIR193A is a popular miRNA in scientific research and stands out for its divergent expression change in CRC vs. IBD relative to LINC01106 as well as its mRNA targets in the core ceRNA network. MIR193A, located on 17q11.2, has been widely found to be dysregulated in tumor progression. Lower expression of MIR193A was observed in human aldosterone-producing adrenocortical adenomas, and was functioned as a tumor suppressor by targeting and restraining CYP11B2 expression both at mRNA and protein level (Zhang et al., 2018). Another group demonstrated that MIR193A played an important role in the regulation of gastric cancer. Both gastric cell lines and human gastric tumors contained decreased levels of MIR193A. In addition, an *in vitro* study showed that upregulating MIR193A decreased tumor cell proliferation and migration, at least partly, by directly targeting cyclin D1 (CCND1) and ETS proto-oncogene 1 (ETS1) expression (Chou et al., 2018). Moreover, LINC00152, a lncRNA overexpressed in gastric cancer, was proved to sponge MIR193A to increase MCL1 expression thereby promoting cells proliferation (Huang et al., 2018). In ovarian cancer, MIR193A is silenced by methylated modification and down regulation of MIR193A expression promotes tumor aggressiveness by losing function of targeting GRB7 and MAPK/ERK pathways (Chen et al., 2018). Similar results were gained by another research that MIR193A is downregulated in metastatic prostate cancer and is involved in HOTAIR/EZH2/miR-193a feedback loop to exert a tumor suppressive function in prostate cancer (Ling et al., 2017). But, contradictorily, MIR193A increases the ability of chemoresistance in prostate cancer cell. MIR193A is noticed to be upregulated in prostate cancer tissues and cell lines, with significant suppression of cell apoptosis induced by oxidative stress by targeting Bach2 (Yang et al., 2017). The role of MIR193a in colorectal cancer and inflammatory bowel disease has also been broadly explored. In 2014, researchers demonstrated that MIR93A is downregulated in CRC to promote lymph node metastasis and result in poor survival (Zhang et al., 2014). In addition, MIR193A was evidenced to target and down regulate colonic PepT1, which reduced intestinal inflammation in response to microbiota (Dai et al., 2015). Another study confirms the tumor suppressor roles of MIR193A by perturbing KRAS function in colorectal adenocarcinoma, including reducing cell proliferation, increasing apoptosis, and inhibiting epithelial-mesenchymal transition (Mamoori et al., 2018). Moreover, MIR193A is revealed to be downregulated in ulcerative colitis neoplasia and promote tumorigenesis by releasing its control for IL17RD expression (Pekow et al., 2017). Considering the important roles of miRNAs in the development of CRC, we propose that our future research should therefore focus on the LINC01106-associated pathway and potential miRNA targets, especially MIR193A.

Our current study is based on published GEO datasets and TCGA COAD dataset. A limitation of the research is that we failed to enroll relevant mRNA, lncRNA, and microRNA by comparing IBD-induced CRC and IBD directly since it’s hard to find qualified data. We are trying to collect such kind of samples from clinical patients and will do the validation research in the future.

## Conclusions

The present study identified a series of IBD-related mRNAs and lncRNAs that were potential mediators in the disease development from IBD to CRC. Using a series of bioinformatics analyses, lncRNA LINC01106, DPP10-AS1, and DIO3OS were identified, which were strongly associated with IBD-related CRC. By constructing a ceRNA network with lncRNAs, miRNAs, and mRNAs, we identified the links between these three RNA species. Further functional analyses suggested a potential mechanism through which these ceRNAs mediated tumorigenesis. Our results emphasized the important role of ceRNAs in the pathogenesis of IBD-related CRC, although more research is necessary to confirm our findings.

##  Supplemental Information

10.7717/peerj.7451/supp-1 Table S1 DEGs of CRC vs. IBD comparison in GSE10714

10.7717/peerj.7451/supp-2 Table S2 DEGs of IBD vs. Normal comparison in GSE10714

10.7717/peerj.7451/supp-3 Table S3 DEGs of CRC vs. IBD comparison in GSE4183

10.7717/peerj.7451/supp-4 Table S4 DEGs of IBD vs. Normal comparison in GSE4183

10.7717/peerj.7451/supp-5 Table S5 DEGs of CRC vs. Normal comparison in TCGA COAD

10.7717/peerj.7451/supp-6 Table S6 DE-miRNAs of IBD vs. Normal comparison in GSE68306

10.7717/peerj.7451/supp-7 Table S7 DE-miRNAs of Neoplasia vs. IBD comparison in GSE68306

10.7717/peerj.7451/supp-8 Table S8 DE-miRNAs of CRC vs. Normal comparison in TCGA COAD

10.7717/peerj.7451/supp-9 Table S9 Expression data of DEGs in heatmap

10.7717/peerj.7451/supp-10 Table S10 The connection information in the total ceRNA network

10.7717/peerj.7451/supp-11 Table S11 Expression data of ceRNA network nodes according to CRC vs. IBD comparison

10.7717/peerj.7451/supp-12 Table S12 Information of PPI network nodes

10.7717/peerj.7451/supp-13 Table S13 List of TF targeted DEGs

10.7717/peerj.7451/supp-14 Figure S1 Data quality analyses of gene expression (A), (B) and (C): Distribution of gene expression levels in each group. (D), (E) and (F): Distribution of genes expression for each sample. QC, quality control.

10.7717/peerj.7451/supp-15 Figure S2 The identification of differential expressed genes in comparisons of CRC vs. Normal (A–C): Volcano graph displaying pairs of expressed genes.

## Abbreviation

DEG: Differential expressed gene
CRC: Colorectal cancer
IBD: Inflammatory bowel disease
HR: Hazard ratio
UC: Ulcerative colitis
CD: Crohn’s disease
ceRNA: Competing endogenous RNA
lncRNA: Long non-coding RNA
GO: Gene ontology
KEGG: Kyoto Encyclopedia of Genes and Genomes
TF: Transcription factor

## Additional Information and Declarations

### Data Availability

The following information was supplied regarding data availability: Data is available at The Cancer Genome Atlas via XENA (https://xenabrowser.net/datapages/?host=https%3A%2F%2Ftcga.xenahubs.net&removeHub=https%3A%2F%2Fxena.treehouse.gi.ucsc.edu%3A443) and GEO (GSE10714, GSE4183, and GSE68306).
