# Supplementary figures and images for: Profiling the lncRNA-miRNA-mRNA ceRNA network to reveal potential crosstalk between inflammatory bowel disease and colorectal cancer
Source: PeerJ. 2019 Aug 26;7:e7451. doi: 10.7717/peerj.7451 (PMC6714963; doi:10.7717/peerj.7451)

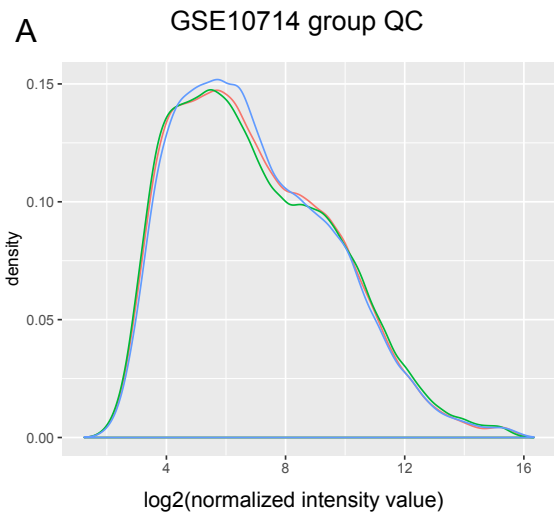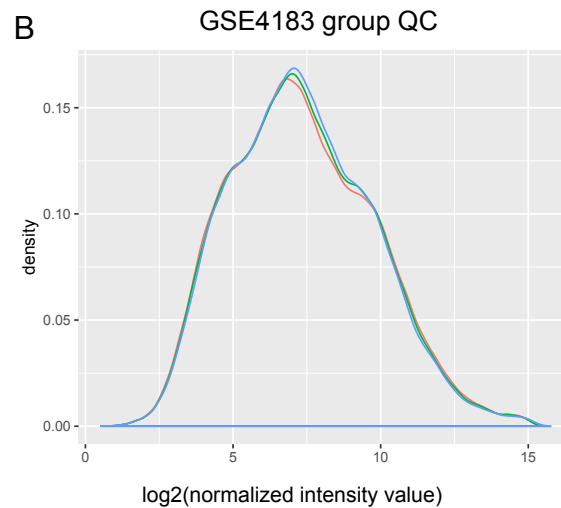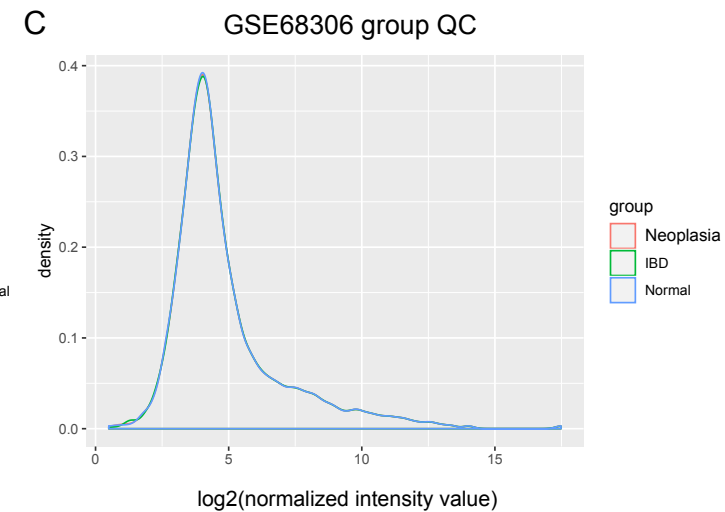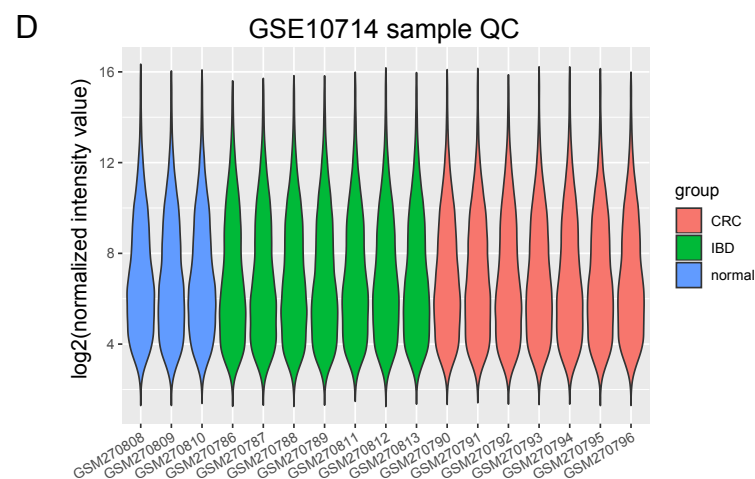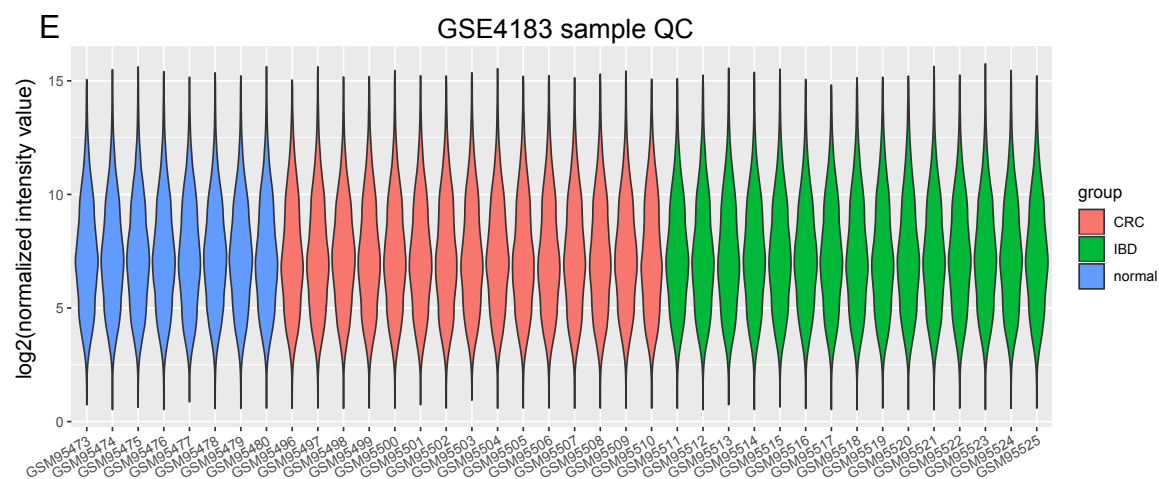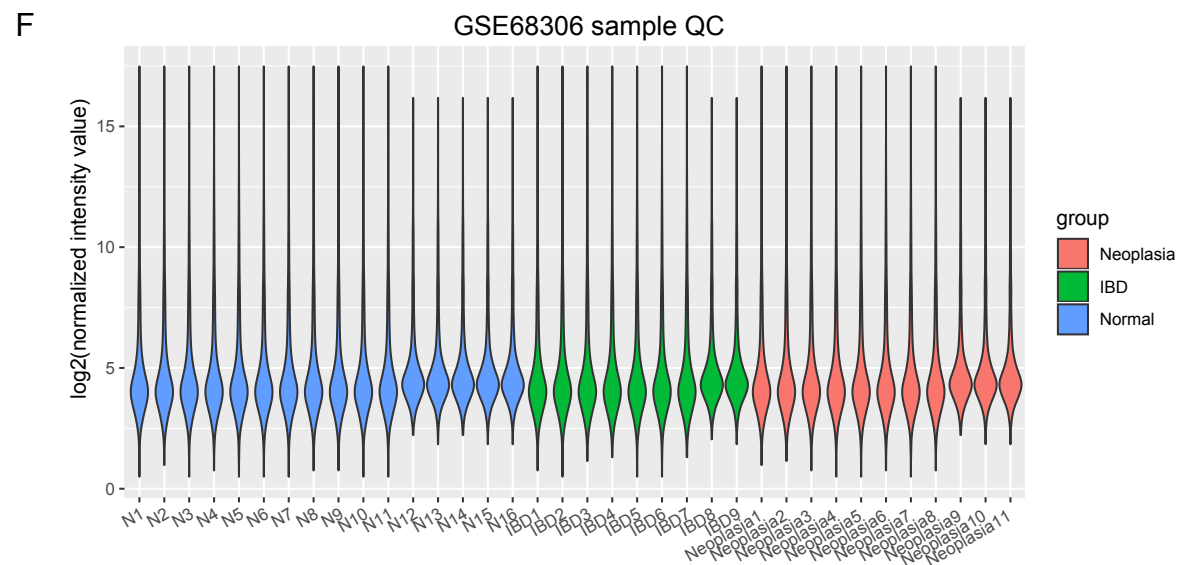

Supplement: Figure S1 — (A), (B) and (C): Distribution of gene expression levels in each group. (D), (E) and (F): Distribution of genes expression for each sample. QC, quality control. [file peerj-07-7451-s014.pdf]
