# Supplementary material for: Profiling the lncRNA-miRNA-mRNA ceRNA network to reveal potential crosstalk between inflammatory bowel disease and colorectal cancer
Source: PeerJ. 2019 Aug 26;7:e7451. doi: 10.7717/peerj.7451 (PMC6714963; doi:10.7717/peerj.7451)
Supplement: Figure S2 — (A–C): Volcano graph displaying pairs of expressed genes. [file peerj-07-7451-s015.pdf]

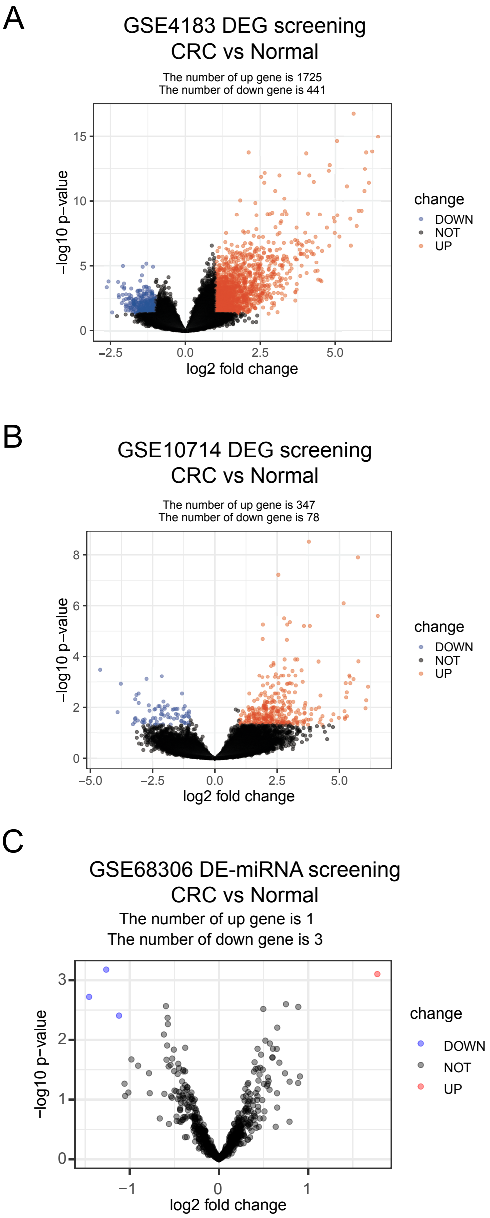
A
GSE4183 DEG screening
CRC vs Normal
The number of up gene is 1725
The number of down gene is 441
−log10 p−value
log2 fold change
change
DOWN
NOT
UP
B
GSE10714 DEG screening
CRC vs Normal
The number of up gene is 347
The number of down gene is 78
−log10 p−value
log2 fold change
change
DOWN
NOT
UP
C
GSE68306 DE-miRNA screening
CRC vs Normal
The number of up gene is 1
The number of down gene is 3
−log10 p−value
log2 fold change
change
DOWN
NOT
UP
